# Using an acellular dermal matrix as a neuroprotective wrap-around for treating recurrent nerve entrapment syndromes: a proof of concept

**DOI:** 10.1007/s10561-025-10159-2

**Published:** 2025-02-05

**Authors:** Till Wagner, Dietmar J. O. Ulrich

**Affiliations:** Department of Plastic and Reconstructive Surgery, Radboud University Medical Center Nijmegen (Radboudumc), Geert Grooteplein, 10, 6525 GA Nijmegen, The Netherlands

**Keywords:** Nerve entrapment syndrome, Carpal tunnel syndrome, Acellular dermal matrix, Recurrent nerve compression syndrome, Extracellular scaffold, Glyaderm, Tarsal tunnel syndrome

## Abstract

Recurrent nerve entrapment syndrome is a well-known complication in peripheral nerve surgery that often leads to multiple reoperations and increases the risk of unfavorable outcomes due to scarring. In our outpatient clinic, we found two patients with recurrent nerve entrapment syndrome with significant symptoms such as complete sensory loss and chronic pain who were willing to undergo re-exploration of their entrapped nerves and cover them with a human acellular dermal matrix (ADM) as a protective shield against recurrence. Both patients had complete recovery of the nerve entrapment syndrome with satisfactory clinical results. The use of a human ADM appears to be a promising tool for recurrent nerve entrapment surgery without adding morbidity to the procedure.

## Introduction

Recurrent nerve entrapment is a well-known complication after surgical nerve decompression. Revision surgery in recurrent carpal tunnel syndromes is low but still exists (1,5% up to 3%−12%)(Chrysopoulo et al. 2006; Stütz et al. 2006; Pripotnev and Mackinnon 2022). The percentage of recurrence or even reoperations in tarsal tunnel syndrome is estimated between 3 and 19% (Ence and DeGeorge 2023). Multiple different treatment options are published for recurrent carpal tunnel syndrome like hypothenar fat pad flaps (Chrysopoulo et al. 2006), local muscle flaps, muscle/skin/synovial flaps as distant flaps (Uemura et al. 2020; Hutting and Uchelen 2018), or even free flaps like omental flaps (Goitz and Steichen 2005) or others to give extra coverage of the decompressed nerve to avoid or at least minimize recurrent scaring which often leads to recurrence (Hunter 1991). In tarsal tunnel release, well-know options in secondary nerve entrapment surgery are bovine collagen tubes as conduits or the use of a greater saphenous vein as a wrap-around (Gould 2014). The drawback of the use of autologous veins as a wrap-around is the extra donor site morbidity and the risk of losing a donor for cardiac bypass graft surgery. The drawbacks of bovine collagen tubes or others are high costs, the absence of elastin (Mayrhofer-Schmid, et al. 2023), the potential risk of allergic reactions caused by immunogenicity, and the limited length available for shielding. Animal studies showed decreased inner epineural connective tissue formation with nerve wrapping with collagen tubes(Kim et al. 2010) in acute nerve lesions or reduced scaring with autogenous vein grafts(Xu et al. 2000). In literature, there is one publication of nerve wrapping with an ADM for nerve protection in chronic complete proximal hamstring ruptures and ischial apophyseal avulsion fractures (Haus et al. 2016). Our idea was to use a wrap-around human acellular dermal matrix as a neuroprotective shield for the treatment of recurrent nerve entrapment syndromes. Extracellular dermal scaffolds have been widely used in breast and abdominal wall surgery and are a well-known and reliable clinical tool.

## Methods and patients

We selected two patients in our outpatient department with recurrent nerve entrapment syndrome. After an extensive explanation of the scheduled procedure, both patients gave informed consent for a rerelease of the entrapped nerve in combination with an acellular derma matrix as a wrap-around for neuroprotection and reducing postop scaring as a proof of concept treatment. The first patient, a 58-year-old right-dominant diabetic male, referred from the department of neurology of our university hospital- presented with a bilateral recurrent carpal tunnel syndrome with four times previous release procedures elsewhere on the left hand and two times on the right hand. The last operation on the left side was affected by a wound infection – a hand phlegmon with consecutive reexploration and a secondary intention to heal, which resulted in the worsening of his complaints. On ultrasound, carpal tunnel syndrome was visible on both hands. With an EMG-proven carpal tunnel syndrome, we scheduled him for rerelease with microscopic neurolysis and neuro-wrapping with Glyaderm as an extra protective barrier against scaring or for a hypothenar fat pad flap, if possible. During the procedure, we found a scarred median nerve without the option to cover it with a local fat flap. Therefore, we covered the whole median nerve within the carpal tunnel after microscopic neurolysis with a strip of meshed ADM (Glyaderm), sutured with three Vicryl 4/0 interrupted sutures to prevent scar contraction. We advise a minimum of 10 min of rinsing before insertion according to the manufacturer’s instructions as described in the user’s manual.

**Fig. 1:**
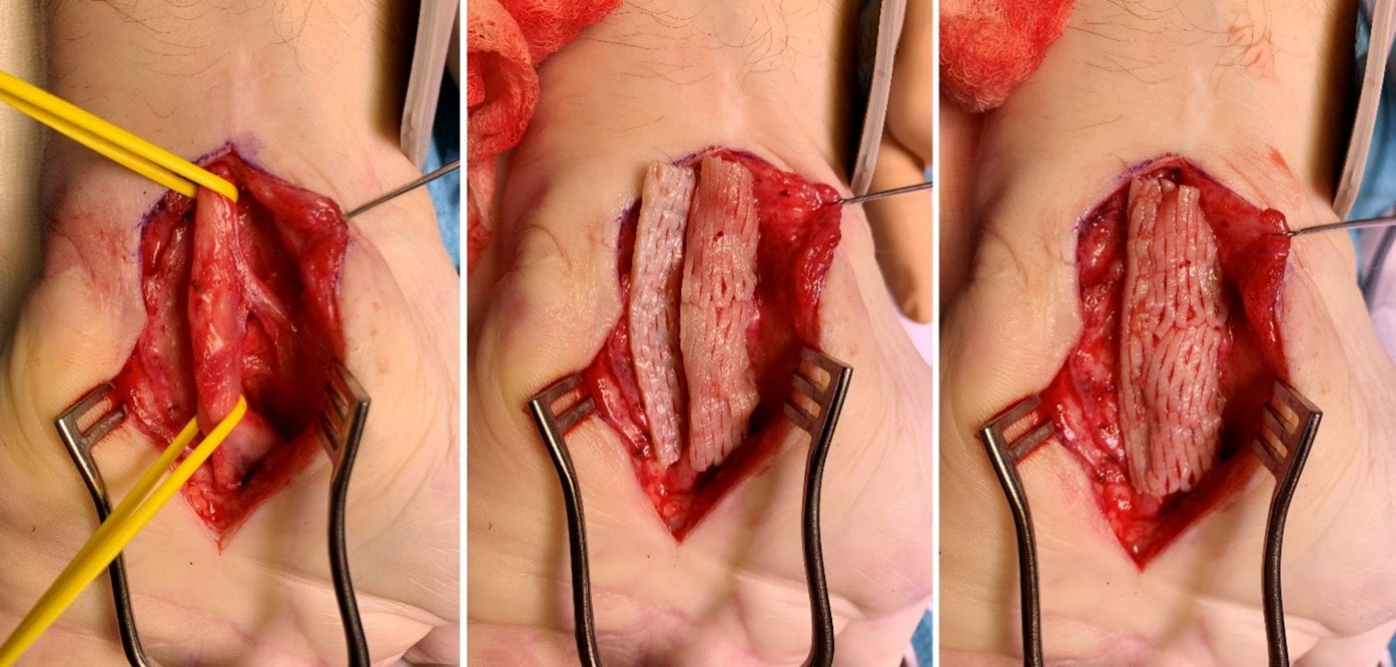
Patient 1 with intraoperative neurolysis and nerve wrapping with meshed Glyaderm on his left hand

**Fig. 2:**
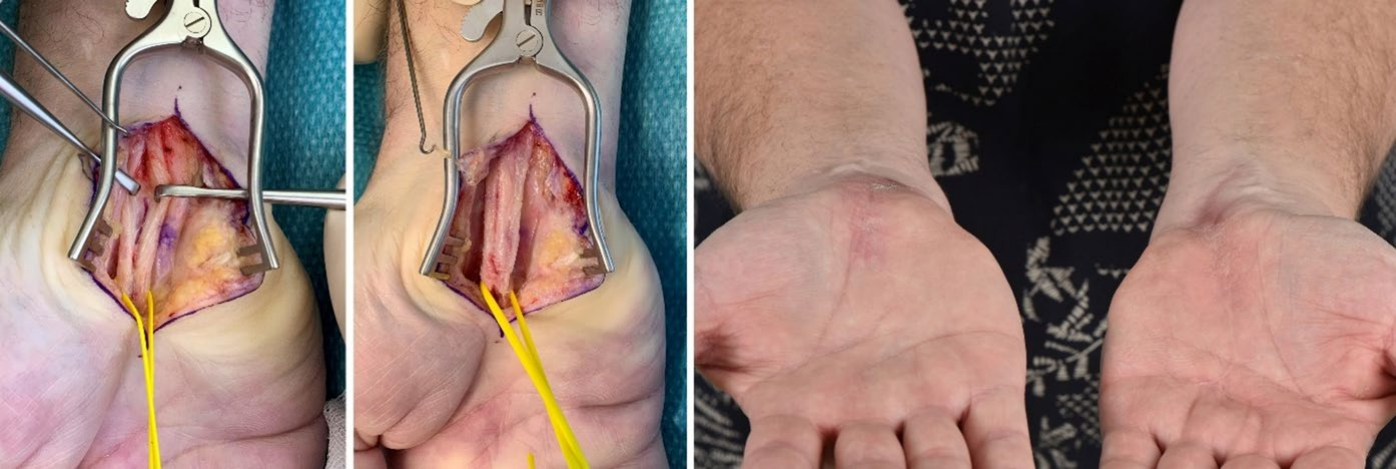
Same patient with carpal tunnel release, dissection of the median nerve because of a median artery and neuro-wrapping with Glyaderm on his right hand and postop results

Further clinical course was uneventful. After six months, the patient was symptom-free, satisfied with his result, and asked for the same procedure on his right side. We did the same procedure -with the additional finding of a median artery, which we resected- also successfully, and the patient returned three months later on control without any further complaints and satisfied (Figs. 1 and 2) with a VAS of 0.

**Fig. 3:**
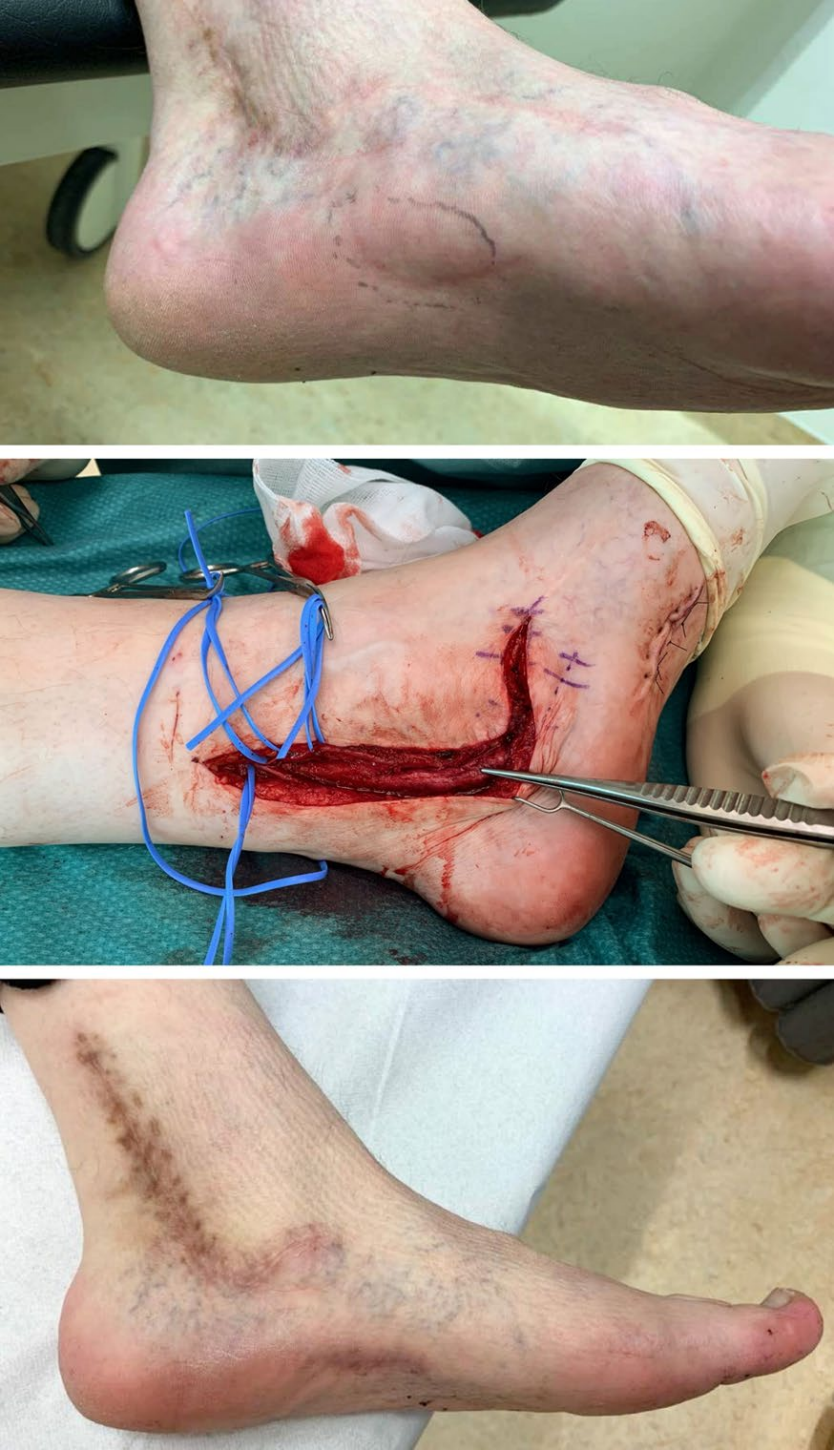
Second patient with tarsal tunnel release and also Glyaderm cover with pre- and postoperative photos

The second patient – a 49 years old, further healthy male- was referred from our hospital’s chronic pain team to our department with a recurrent tarsal tunnel syndrome with chronically untreatable pain −9 out of 10 on VAS during activity- on the medial aspect of his plantar foot and retro-malleolar, which forced him to use a wheelchair and crutches, because walking was impossible. He was treated ten years ago elsewhere for a common tarsal tunnel syndrome. We scheduled him for rerelease of his tarsal tunnel syndrome with neurolysis of his medial plantar nerve via an additional incision. We performed a microsurgical neurolysis of the fibrotic nerve, which was intact without any neuromas. We covered the nerve with Glyaderm in a wrap-around technique over the complete length of the tarsal tunnel. The Glyaderm scaffold has been sutured with three Vicryl 4.0 sutures. The skin was closed in both patients in a standard fashion. The further clinical course was uneventful, and the progress was postoperatively satisfying. The patient returned nine months later on control full ambulatory, without any complaints (VAS 0), and had restarted working (Fig. 3).

## Discussion

Recurrent nerve entrapment syndromes are a well-known issue in peripheral nerve surgery. In most cases, scarring or adherence is one of the main reasons for recurrence. Thus, several options have been published to prevent or to reduce recurrent scarring (Pripotnev and Mackinnon 2022; Masear 2011). We selected two patients from our outpatient department with chronic pain otherwise unable to treat. We have chosen Glyaderm with its human origin to reduce the possibility of allergic or immunogenic reactions (Henau et al. 2021; Pirayesh et al. 2015) and the high percentage of elastin, which has proven to suppress the differentiation and proliferation of fibroblasts in contractile myofibroblasts. Glyaderm (Glycerol preserved Acellular Dermis) is also cost -efficient with 4.60 Euro/cm2 in 2020, is produced by a non-profit organization (Euro Tissue Bank ETB-BISLIFE, Skin department, Beverwijk, The Netherlands) (Decker, et al. 2023; Pirayesh et al. 2022). Glycerol preservation and NaOH incubation of all antigenic structures and cells are removed and meshed for more elasticity. It was easy to obtain because of its regular use in our plastic surgery children’s trauma and burn unit as a dermal skin substitute. Additional value of Glyaderm is the low antigenicity of the human acellular dermal matrix and its high biocompatibility. Acellular dermal matrices of scaffolds are known to reduce scarring in abdominal wall reconstruction (Petrie et al. 2022) and to reduce capsule contractions in breast reconstructive surgery (Macadam and Lennox 2012). Another advantage compared to known treatment options is zero extra donor-site morbidity and the easy use in the meaning of length and width (longer than the commonly available 5 cm length in different scaffolds), thus easily individually tailorable to the patient’s needs. Our publication is the first report of successfully treating a recalcitrant nerve entrapment syndrome in humans with an extracellular scaffold with satisfactory clinical outcomes. It is unknown if this is an effect solely of Glyaderm or also with other alloplastic/xenoplastic ADMs. Further research has to be done to delineate the clinical value of this treatment option.

## Conclusions

Concluding our first clinical findings, Glyaderm seems to be a new promising tool for recurrent nerve entrapment syndromes, but more prospective research should be done.

## Data Availability

No datasets were generated or analysed during the current study.
